# Prenatal and early-life diesel exhaust exposure causes autism-like behavioral changes in mice

**DOI:** 10.1186/s12989-018-0254-4

**Published:** 2018-04-20

**Authors:** Yu-Chi Chang, Toby B. Cole, Lucio G. Costa

**Affiliations:** 1 Department of Environmental and Occupational Health Sciences, University of Washington, Seattle, Washington USA; 2 Center on Human Development and Disability, University of Washington, Seattle, Washington USA; 3 Department of Medicine and Surgery, University of Parma Medical School, Parma, Italy

**Keywords:** Autism, Air pollution, Diesel exhaust, Mice, Behavior

## Abstract

**Background:**

Escalating prevalence of autism spectrum disorders (ASD) in recent decades has triggered increasing efforts in understanding roles played by environmental risk factors as a way to address this widespread public health concern. Several epidemiological studies show associations between developmental exposure to traffic-related air pollution and increased ASD risk. In rodent models, a limited number of studies have shown that developmental exposure to ambient ultrafine particulates or diesel exhaust (DE) can result in behavioral phenotypes consistent with mild ASD. We performed a series of experiments to determine whether developmental DE exposure induces ASD-related behaviors in mice.

**Results:**

C57Bl/6J mice were exposed from embryonic day 0 to postnatal day 21 to 250–300 μg/m^3^ DE or filtered air (FA) as control. Mice exposed developmentally to DE exhibited deficits in all three of the hallmark categories of ASD behavior: reduced social interaction in the reciprocal interaction and social preference tests, increased repetitive behavior in the T-maze and marble-burying test, and reduced or altered communication as assessed by measuring isolation-induced ultrasonic vocalizations and responses to social odors.

**Conclusions:**

These findings demonstrate that exposure to traffic-related air pollution, in particular that associated with diesel-fuel combustion, can cause ASD-related behavioral changes in mice, and raise concern about air pollution as a contributor to the onset of ASD in humans.

**Electronic supplementary material:**

The online version of this article (10.1186/s12989-018-0254-4) contains supplementary material, which is available to authorized users.

## Background

Autism spectrum disorders (ASD) represent a heterogeneous group of disorders characterized by three behavioral domains: difficulties in social interactions, issues with verbal and nonverbal communication, and repetitive behaviors [1]. According to a 2010 survey by the U.S. Centers for Disease Control and Prevention, autism prevalence has been reported to be 1 in 68 children in the United States, indicating a ten-fold increase in the past 40 years. Only a small subgroup of ASD cases can be attributed to genetics alone [2], and environmental factors are believed to contribute substantially to ASD etiology. Increased ASD risk has been associated with various environmental exposures such as air pollution, heavy metals, organophosphorus insecticides, perinatal stress and infectious agents [3–13]. Increasing prevalence in recent years could be attributed to both broadening of diagnostic definition and increased exposure to environmental toxicants. An epidemiological study conducted in California estimated that 26.4% of the increased autism prevalence can be attributed to change in diagnostic practices between 1992 and 2005 [14]. Adding to the significance of environmental contribution to autism etiology, an epidemiological study by Hallmayer et al. [15]*,* looking at ASD association in monozygotic and dizygotic twins, concluded that environmental components have a larger effect than genetic components in predicting ASD outcome.

Traffic-related air pollution (TRAP) is a widespread environmental concern, especially in densely populated areas such as Central America and South and East Asia [16, 17]. High exposure levels of particulate matter (PM > 100 μg/m^3^) over extended periods have been commonly experienced by populations living in these areas [18]. Air pollution is a mixture of several components, including gases, organic compounds, metals, and ambient PM. The sources of particulate air pollution, and hence its composition, can vary greatly from region to region, and diesel exhaust (DE) is an important, but not the only, component. In urban areas where traffic-related air pollution is of particular concern, a large proportion of utility vehicles operate on diesel fuel, including vehicles used for public transportation or transportation of commercial cargo, as well as many personal passenger vehicles. For example, at least 1/3 of the total road transport fuel consumption in the United Kingdom between 2010 and 2012 was due to consumption of diesel fuel [19]. In addition, data collected in 2010 in Mexico City showed that DE has been estimated to contribute > 35% of ambient PM2.5 [20]. It should be also mentioned that new filtration technologies, which are being increasingly utilized, significantly reduce the release of PM. However, such measures may not be available or used in all parts of the world, leading to high PM2.5 levels from DE and other sources.

Developmental exposure to TRAP has been associated with increased ASD risk in many recent epidemiological studies conducted by research groups in North America and Europe [4, 5, 21–24]. Two studies by Volk et al. [5, 24] reported that children exposed to higher levels of TRAP due to residential proximity to busy roadways are at a higher risk of developing ASD. Another study by Roberts et al. [21] also reported increased ASD risk with diesel particulate exposure. Similarly, increased ASD risk with TRAP has been reported in populations in Taiwan and in Pennsylvania [25, 26]. Epidemiological studies conducted as part of the Nurses’ Health Study II Cohort revealed the strongest association between ASD risk and TRAP when exposure occurred during late gestation and the neonatal period [5, 24, 27, 28]. The third trimester brain growth spurt has been identified as a particularly vulnerable period of brain development in studies focused on developmental neurotoxicity of alcohol. In rodent models, hippocampal seizures, depression, and persistent cognitive effects were found when ethanol exposure occurred during this peak period of brain growth [29, 30]. On a related note, air pollution exposure has been shown to cause a decrease in cortical thickness and lateral ventricular size in rodent models [10, 31], suggesting that this period of intense brain growth in the third trimester may be an important window of vulnerability for neurodevelopmental toxicity related to TRAP as well. In rodents, this window of particularly robust brain growth extends into the postnatal period [32, 33].

The few available animal studies also suggest a connection between developmental exposure to air pollution and ASD. Mice exposed developmentally to high levels of DE particles exhibited altered behavioral phenotypes, including effects on locomotor activity and repetitive behaviors [34]. Mice exposed perinatally to ultrafine ambient particles exhibited repetitive and impulsive behaviors, as well as ventriculomegaly, a brain structural morphological change also reported in some ASD patients [10, 35]. Additionally, prenatal exposures of mice to low levels of DE or DE particles have been shown to result in altered locomotor activity [36]. While epidemiological studies suggest an association between elevated air pollution and ASD, it is difficult to control for several potential confounders, and controlled-exposure animal studies have so far provided information only on a subset of autism related behavior phenotypes. The purpose of the present study was to conduct a more thorough evaluation of ASD-related behavioral endpoints, upon developmental exposure of mice to environmentally-relevant levels of DE under a controlled exposure setting.

## Methods

### Animals and overall study design

All animal experiments were approved by the University of Washington Institutional Animal Care and Use Committee, and performed according to the National Research Council Guide for the Care and Use of Laboratory Animals, as adopted by the National Institutes of Health. C57Bl/6J mice of both sexes (Jackson Laboratory, Bar Harbor, ME) were housed in University of Washington centralized vivaria under specific pathogen free (SPF) conditions, on a 12 h light/dark cycle. Mice were housed in Allentown housing racks using 42 cm (L) X 42 cm (W) X 20 cm (H) acrylic cages supplied with water bottles, food hoppers, cotton nestlets, and acrylic huts. Air (HEPA-filtered room air or diluted DE as described below) was provided to individual cages via the air circulation system in the Allentown racks. Before and during pregnancy and throughout the preweaning period, mice were provided with breeder chow (Picolab irradiated mouse diet 5085; LabDiet, St. Louis, MO). After weaning, mice were provided with standard rodent chow (Picolab irradiated rodent diet 5053; LabDiet, St. Louis, MO). Before the onset of the experiments, mice were acclimated for at least 1 week, with daily handling, to the vivarium at the UW Controlled Exposure Laboratory’s Northlake Diesel Facility.

**Fig. 1:**
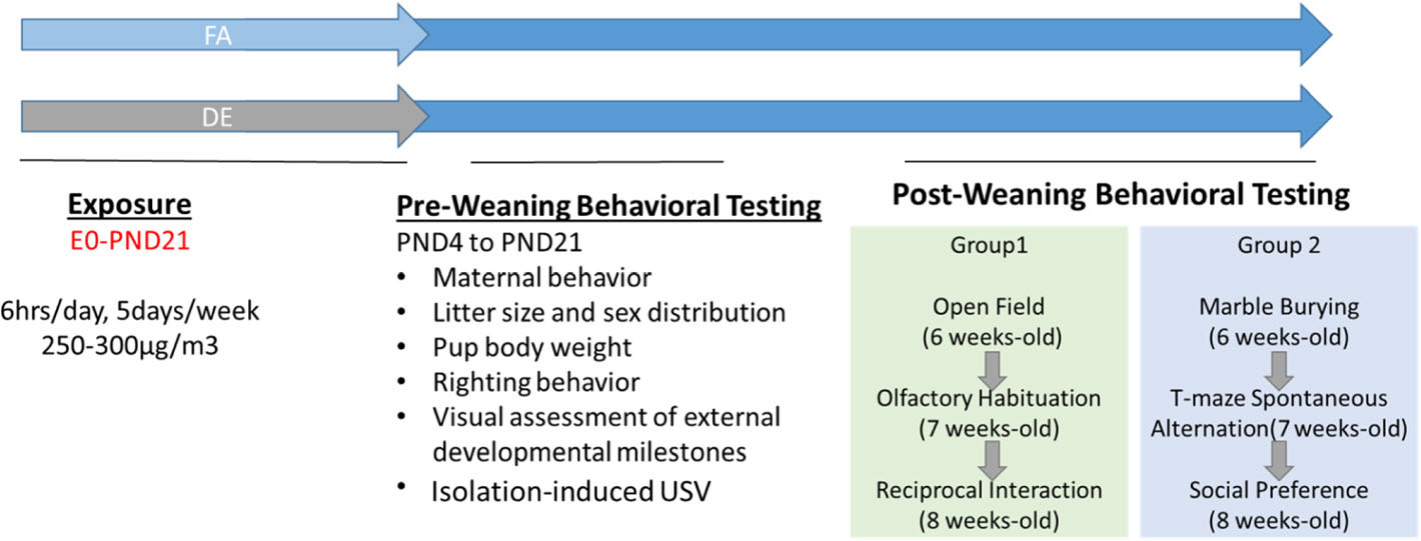
Experimental Design. C57/BL6J mice were time-mated and exposed to 250–300 μg/m^3^ of DE or FA from E0 to PND21 for 6/day and 5 days/week. Pre-weaning developmental assessment started on PND3. Behavioral assessment focusing on three characteristic domains of ASD started when the mice were 6 weeks old

The overall study design, including the exposure and behavioral testing timelines, is shown in Fig. 1. Pregnant dams and pups were exposed to DE (250–300 μg/m^3^ PM concentration) for 6 h per day and 5 days per week (Monday – Friday) from embryonic day 0 (E0) to postnatal day (PND) 21, as detailed below. The exposure period was based on epidemiological studies in humans showing that traffic-related air pollution exposure during all three trimesters of pregnancy and during the first 9 months of infants’ life is associated with increased ASD risk [5, 22, 24, 27]. The exposure duration was designed to cover key neurodevelopmental events happening during this window of susceptibility, which in mice equates to the period from E0 to PND21 [33]. For exposure of pregnant dams and pups, female mice (10–13 wk-old) were time-mated with males (3 females per breeding cage) on Sunday evenings, and copulatory plugs were checked the next morning before the Monday onset of the weekly exposures. Mice with confirmed vaginal plugs were removed from the breeding cage on E0 (on the morning of vaginal plug confirmation) and housed individually in cages in either the FA or DE housing racks for the duration of pregnancy, parturition, and until weaning of the litter. The use of Sunday timed matings ensured that E0 always occurred on a Monday, and that parturition (PND0) would occur on a Saturday or Sunday. This ensured the same developmental exposure periods for all mice (i.e., all dams were exposed on the same gestational days: E0–4, E7–11, & E14–18), and that DE exposure would not occur while mice were giving birth (on E19 or E20). On PND3, litters were culled to 5 pups, with two male and two female pups from each litter selected randomly for behavioral testing. Excess pups were euthanized by decapitation on PND3, and runts (weight > 15% lighter than their litter mates) were excluded from the study. Mice for behavioral testing were tattoo-marked on their paw pads on PND3, and were weighed individually each day throughout the preweaning period. On PND21, after the last DE exposure, mice were weaned and transferred to standard Allentown housing racks (two same-sex mice per cage) for the duration of the post-weaning behavioral testing period.

### Diesel exhaust (DE) exposures

DE exposures were carried out in the UW Controlled Exposure Laboratory’s Northlake Diesel Facility [37, 38]. DE was generated from a single-cylinder Yanmar diesel generator engine (YDG5500), fueled with standard highway-grade number 2 diesel fuel obtained from local fuel distributors, and operated under ~ 70% electrical load. DE passed from the generator exhaust through a two-step dilution system, with continuous dynamic control of the fine particulate matter (PM2.5) concentration, allowing maintenance of a constant exposure level at 250–300 μg/m^3^ PM monitored with an in-cage nephelometer. Diluted DE was delivered through a ductwork system to the air intake of an Allentown mouse housing rack, which distributed the diluted DE evenly to all of the cages in the rack. Control mice were exposed to filtered air (FA) in a separate Allentown housing rack located in the same facility, which supplied HEPA-filtered room air to the cages via its ventilation system. Chemical composition and particle size characterization of the DE generated in this facility has been described previously in detail [37–39]. Briefly, under identical conditions to those used in the current study, particle size characterization, as measured by gravimetric analysis using a micro orifice uniform deposit impactor (MOUDI) demonstrated a median diameter for in-cage particles of 77 nm, while the count median diameter was 87 nm, indicating a large portion of DE particles in the ultrafine size range [39]. The mass fraction of particle-bound poly-cyclic aromatic hydrocarbons (PAH) was 20 ng/μg PM2.5, with a ratio of organic carbon to elemental carbon mass concentration of 0.10. Under these conditions, the concentrations of oxides of nitrogen in the DE were 1800 ppb NO_x_ and 60 ppb NO_2_, carbon monoxide concentration was 2 ppm, and carbon dioxide concentration was 1000 ppm. Additional characterization of the elemental content of the DE has been described in detail [38–40]. DE exposures for the current study were performed under identical conditions, and real-time particle concentrations were measured continuously using an in-cage nephelometer, with fine-tuning of the exposure level throughout each exposure by manual adjustment of the electrical load (2.0–2.5 Kw) and/or air flow.

### Behavioral testing

Behavioral tests were selected based on relevance to the three diagnostic domains of autism spectrum disorder [41]. Deficit in social interaction was assessed by the social preference test and reciprocal interaction test. Vocal and olfactory communication deficits were assessed by neonatal ultrasonic vocalization (USV) recording, and by the olfactory habituation test. Persistent/repetitive behavior was assessed by the marble burying test and the T-maze spontaneous alternation test. To avoid interference associated with multiple testing, animals undergoing behavioral tests were separated into two testing groups as shown in Fig. 1: one male and one female from each litter were randomly assigned to each of the two behavioral testing groups, and subsequent tests performed on the same subjects were separated by at least 1 week. To assess pregnancy outcome, litter size, sex distribution and duration of pregnancy were recorded for each litter. Pre-weaning assessments, including righting reflex, neonatal USVs and body weights were recorded starting from PND4. Post-weaning behavioral testing was conducted beginning at 6 weeks of age, using the same animals tested during the pre-weaning period. All behavioral tests were performed in the daytime during the light cycle. At the conclusion of behavioral testing (10-wk), mice were euthanized by CO_2_ asphyxiation followed by cervical dislocation.

#### Maternal behavior

Maternal behaviors were scored from PND0 to PND2, once per day for 30-min, between 6:00–10:00 AM. During each scoring session, behaviors such as eating, drinking, pup grooming, arch-back feeding, blanket feeding, nest building, digging, and rearing were scored every minute. The maternal behavior was stratified into behaviors that were related to pup care (feeding, pup grooming, nest building) and behaviors that were not related to pup care (eating, drinking, self-grooming, rearing). Quality of pup care was quantified as the percentage of time the dam spent on activities relating to pup care during the 30 min scoring session.

#### Righting reflex

Righting reflex was measured on PND4–9 and PND12; the pups were placed on a flat clean paper towel dorsal side down, and latency to resume righting position (all four paws on the ground) was measured. Each trial was allowed a maximum of 30 s to complete. Mice acquiring the reflex were retested on all subsequent days.

#### Open field test

Each mouse was placed into a clean 42 cm (L) X 42 cm (W) X 20 cm (H) acrylic cage with no bedding for 30 min and was video-recorded (Microsoft LifeCam HD-6000). Locations of subjects were tracked with Ethovision XT 11 (Noldus Information Technology, Leesburg, VA - USA) to assess anxiety response in a novel environment. Time spent in the periphery area (thigmotaxis) versus center area (20 X 20 cm) was measured automatically by the Ethovision software.

#### Social preference test

The social preference test was conducted using a three chambered social approach apparatus, a clear acrylic box consisting of three same-sized chambers (20 cm × 40 cm × 22 cm) with small openings in the dividing walls that allowed the subject to access all three chambers without restriction. A metal-wire holding cup for the novel and familiar mice was placed in each of the side chambers. The testing procedure consisted of three chronological phases: habituation, sociability, and social novelty [41]. Additional file 1: Figure S1 illustrates the arena setup for the sociability and social novelty phases. In the habituation phase the test mouse was placed into the middle chamber and allowed to explore all three chambers freely for 10 min. In the sociability phase, an age- and gender-matched novel C57Bl/6J mouse was placed into one of the holding cups. Sociability was quantified as the amount of time the test mouse spent in proximity (inside of the interaction zone, within a 5 cm radius from the holding cup) to the holding cup containing the first novel mouse versus the time spent in proximity to the empty holding cup. In the social novelty phase, a second age- and gender-matched novel mouse was added into the holding cup that had been empty during the sociability phase. The preference of the test mouse for social novelty was quantified by measuring time spent in the interaction zone near the second novel mouse versus time spent in the interaction zone near the now-familiar mouse.

#### Reciprocal Interaction

Reciprocal interactions between the test subject and an age- and sex-matched socially naïve C57Bl/6J stimulus partner were assessed [41]. Test and naïve mice were individually housed for an hour before being introduced to each other in a clean standard housing cage for 10 min. A Microsoft LifeCam HD-6000 camera was used for video recording. Total social contact, including sniffing (nose–nose, anogenital, body), push–play behavior, following, rearing, and huddling was scored manually by a researcher blinded to the experimental groups.

#### Olfactory habituation to social odors

For olfactory communication, sniffing response toward different olfactory cues with and without social valence was assessed [41]. Mice were housed individually for an hour before testing began. Olfactory cues were prepared in the following concentrations: distilled water, almond extract (McCormick, Hunt Valley, MD; 1:100 dilution), banana flavoring (McCormick, Hunt Valley, MD; 1:100 dilution), pooled male urine (from age-matched C57Bl/6J mice at 1:100 dilution), and pooled female urine (from age-matched C57Bl/6J mice at 1:100 dilution). For presentation, 10 μl of olfactory stimuli were applied onto filter paper taped inside of polystyrene weigh boats (VWR, Radnor, PA; 4.1 × 4.1 × 0.8 cm) and placed on top of the wire cage-top for 2 min before replacing with the next olfactory stimulus. During each 2 min presentation period, test-mouse behavior was video-recorded (Microsoft, LifeCam HD-6000). Number of sniffs and duration of sniffs were scored manually from the video-recording by two different researchers who were blinded to the treatment groups.

#### Neonatal isolation-induced ultrasonic vocalization

To assess neonatal vocal communication, ultrasonic vocalizations (USV) were recorded for 5 min from PND6 mice isolated from their dam and littermates inside of a 250 ml glass beaker and surrounded by a sound-attenuating box, with an electrical heating pad (Sunbeam 731–500) maintaining temperature at 22–25 °C. USVs were recorded with the UltraVox system from Noldus Information Technology (Leesburg, VA, USA). Frequency and duration of calls were quantified in ten call categories: 1) complex, 2) two-components, 3) upward, 4) downward, 5) chevron, 6) short, 7) composite, 8) frequency-steps, 9) flat, and 10) unstructured calls, as shown in Additional file 1: Figure S3, using the UltraVox software provided by Noldus Information Technology (Leesburg, VA, USA). Characteristic criteria for each call category were as described by Scattoni et al. [42].

#### Marble burying test

Repetitive digging was assessed by placing the mouse into a clean standard sized housing cage (484 sq. cm) filled with shaved aspen bedding 5 cm in depth then topped with 12 marbles (1.58 cm in diameter) evenly spaced in a 3 X 4 grid. A clear acrylic ceiling with ventilation holes was used to prevent mice from climbing out of the cage during testing. Digging activity was video-recorded for 30 min (Microsoft, LifeCam HD-6000); number of buried marbles (> 2/3rd covered) were scored every 5 min from video footage by a researcher blinded to exposure [41]. The cage and acrylic ceiling were cleaned with 70% ethanol then refilled with new bedding material; marbles were washed with soap, rinsed with water and 70% ethanol and dried for subsequent trials.

#### T maze spontaneous alternation test

To assess repetitive behaviors and/or working memory, each subject was placed into a T-shaped maze (consisting of three black plexiglass 30 X 10 cm arms joined by a 10 X 10 cm center) with the nose pointing away from the center, and was allowed to choose between right and left goal arms throughout a 15-trial session. Once a mouse entered a particular goal arm, it was restricted in that goal arm for 30 s before being returned back to the starting position for the next trial of testing. Subjects that failed to enter a goal arm within 2 min were disqualified from testing. The whole testing procedure was video recorded (Microsoft, LifeCam HD-6000) and scored manually. Spontaneous alternation rate was calculated as the ratio between the alternating choices and total number of choices [41].

### Statistical analysis

Statistical analyses were performed using GraphPad Prism software (GraphPad Software Inc., La Jolla, CA). For most measures, statistical significance was determined by two-way ANOVA using exposure and sex as independent variables, with the Bonferroni correction for multiple comparisons. For the marble burying test, statistical significance was determined by two-way ANOVA with repeated measures, using the Bonferroni correction. For the olfactory habituation test, differences in the initial responses to odor presentation were analyzed by two-way ANOVA with Bonferroni correction, whereas habituation (i.e., the decrease in response with repeated presentation of the olfactory cue) was determined for each of the four groups (DE male, FA male, DE female, FA female) by one-way ANOVA with repeated measures, using Dunn’s test for multiple comparisons. For the three chambered social preference test, time spent in the ‘N1’ versus ‘empty’ chambers (for the social approach phase) or ‘N1’ versus ‘N2’ chambers (for the social novelty phase) were compared within each of the four groups by one-way ANOVA with the Bonferroni correction. Values of *p* < 0.05 were considered statistically significant. Results are expressed as the mean ± SEM.

## Results

### The effect of diesel exhaust on pregnancy outcome and pre-weaning behaviors

**Fig. 2:**
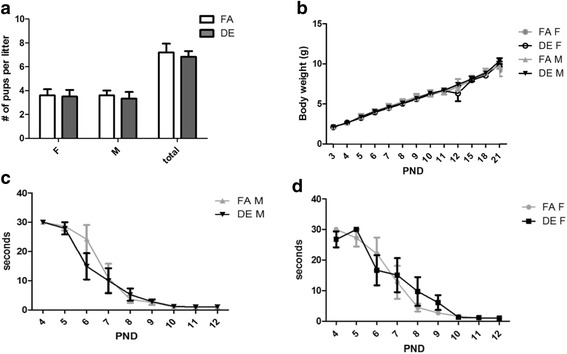
Pre-weaning Developmental Assessment. **a** Number of male, female, and total pups from each litter. No significant differences in sex distribution or number of pups per litter were found between FA and DE groups. (**b**) Pup weight (averaged weights of the 2 same-sex pups from each litter) measured from PND3 to PND21. No significant differences in pup weight between different experimental groups were found. Righting reflex response was measured in males (**c**) and females (**d**) from PND4–12. No significant differences in righting response time (sec) between DE exposed and FA control mice were found in either sex (Two-way ANOVA with Bonferroni correction). FA M *n* = 13, DE M *n* = 14, FA F *n* = 12, DE F *n* = 13

**Fig. 3:**
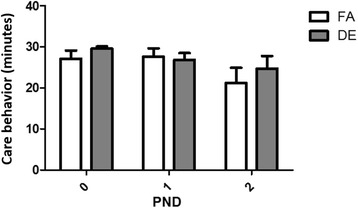
Maternal Care Behavior. Total time (min) spent by dams on behaviors related to caring for pups (pup grooming, nursing, nest building, sitting with pups) was recorded in 1 min intervals for 30 min each day during the first 3 days of pups’ life. No differences were found between FA and DE dams (Two-way ANOVA with Bonferroni correction). FA *N* = 13, DE *N* = 14

For this study 13 FA- and 14 DE-exposed litters were generated over 12 overlapping exposure cohorts. Both DE- and FA- exposed pups were born in similar litter size and sex ratio (Fig. 2a). There were no significant differences in pup weights (Fig. 2b), and the appearance of the righting reflex was the same for both DE- and FA-exposed pups (Fig. 2c, d). Monitoring maternal behavior at PND0–2 revealed no significant differences in time spent on pup-care activities such as grooming, nursing, and nest building between dams exposed to DE or FA (Fig. 3). In the open field test (Additional file 1: Figure S2A, B), no significant differences were found in locomotor activity or thigmotaxis, a possible indicator of anxiety and/or exploratory drive.

### The effect of diesel exhaust on social behavior

**Fig. 4:**
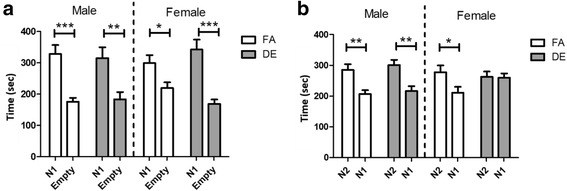
Three Chambered Social Preference Test. In the sociability phase of the three chambered social preference test (**a**), sociability was assessed by measuring cumulative time spent by test mice in chamber containing the novel mouse (N1) vs. empty chamber. There were no differences between FA-exposed and DE-exposed mice; FA and DE-exposed mice of both sexes exhibited preference toward the novel mouse over empty setup (*p < 0.05, **p < 0.01, ***p < 0.001; One-way ANOVA with Bonferroni correction). In the social novelty phase (**b**), cumulative time spent by test mice in the chamber containing the novel mouse (N2) vs. familiar mouse (N1) was measured. DE-exposed female mice showed no preference between the novel vs. familiar mouse, while FA-exposed mice of both sex and DE-exposed males preferred the novel mouse over the familiar mouse (*p < 0.05, **p < 0.01; One-way ANOVA with Bonferroni correction). FA M *n* = 14, DE M *n* = 13, FA F *n* = 13, DE F *n* = 13

In the social preference test, sociability and the ability to detect and remember social novelty were assessed using a three-chambered social approach apparatus. In the sociability phase, DE- and FA-exposed mice of both sexes demonstrated a significant preference for spending time in the chamber containing the novel mouse, compared to the empty chamber (Fig. 4a). The ability to differentiate social novelty was assessed by measuring time spent in the chamber containing a second novel mouse (N2) vs. time spent in the chamber containing the now-familiar mouse (N1). DE-exposed female mice showed no preference between the novel and familiar mice, whereas DE-exposed males and FA-exposed mice of both sexes demonstrated a clear preference for the novel mouse compared to the familiar mouse (Fig. 4b).

**Fig. 5:**
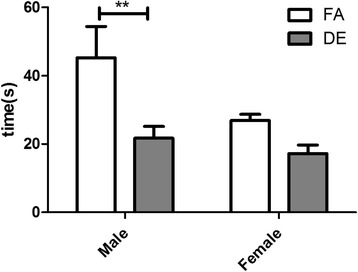
Reciprocal Interaction Test. Interactive sniffing duration (s) during reciprocal interaction was plotted as mean ± SEM. Males exposed to DE exhibited less interactive sniffing compared to FA-exposed control males (**p < 0.01; Two-way ANOVA with Bonferroni correction). DE-exposed female mice also showed a trend of decreased interactive sniffing that was not statistically significant. FA M *n* = 11, DE M *n* = 13, FA F *n* = 10, DE F *n* = 13

The reciprocal interaction test allows assessment of intrinsic social behavior with an age- and sex-matched social partner in an unrestricted setting. During the 10-min interaction period, DE-exposed males spent significantly less time on interactive sniffing with the novel social partner, as compared to FA-exposed males (Fig. 5). No differences were found in interactive sniffing between DE-exposed and FA-exposed females; there was a tendency towards decreased interactive sniffing in the DE-exposed females, but the difference was not statistically significant.

### The effect of diesel exhaust on communication

**Fig. 6:**
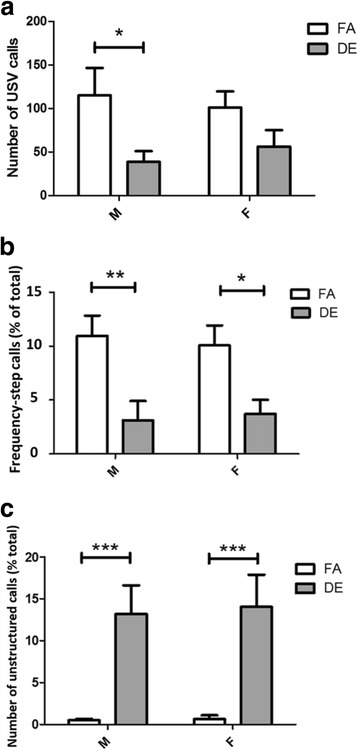
Neonatal ultrasonic vocalizations (USV) on PND6. (**a**) Total number of calls emitted during the 5-min isolation period. Males exposed to DE emitted fewer USV calls compared to FA-exposed control males (*p < 0.05; Two-way ANOVA with Bonferroni correction). (**b**) Frequency-step calls normalized to total number of calls by the same pup. DE-exposed mice of both sex produced fewer frequency-step calls compared to same-sex FA-exposed controls. (Male: **p < 0.01; Female: *p < 0.05; Two-way ANOVA with Bonferroni correction). (**c**) Unstructured calls normalized to total number of calls by the same pup. DE-exposed mice of both sex emitted far more unstructured USV calls compared to same sex FA-exposed controls. (***p < 0.001; Two-way ANOVA with Bonferroni correction). FA M *n* = 16, DE M *n* = 12, FA F *n* = 16, DE F *n* = 12. (See Additional file 1: Figure S3 for examples of each type of USV call)

Vocal communication was assessed by recording isolation-induced neonatal ultrasonic vocalizations (USV) at PND6. DE-exposed males emitted significantly fewer USV calls compared to FA-exposed males (Fig. 6a). DE-exposed females also tended to emit fewer USV calls than FA-exposed females, but the difference was not statistically significant (Fig. 6a). USVs were also categorized into nine USV categories as described by Scattoni et al. [43] (see Additional file 1: Figure S3 for examples of each call category from the current study). Since the number of calls in specific USV categories could be confounded by inter-individual differences in the total number of calls, the number of calls from each USV category was normalized to the total number of calls emitted by the same subject (number of calls emitted by each pup in specific USV category/total number of USV calls). DE-exposed mice of both sexes emitted significantly fewer ‘frequency-step’ calls compared to the FA-exposed mice (Fig. 6b). DE-exposed males and females also emitted significantly more ‘unstructured’ calls compared to the FA-exposed mice (Fig. 6c). Increased emission of unstructured USV calls has also been reported in genetic mouse models of autism [44, 45].

**Fig. 7:**
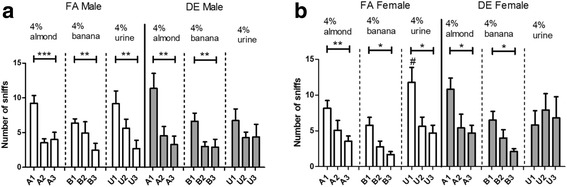
Olfactory Habituation test. In the olfactory habituation test, sniffing responses elicited by repeated presentations of non-social odors (almond and banana) or social odor (same sex urine) were analyzed in males (**a**) and females (**b**). For the initial presentation of same-sex urine (U1), FA-exposed female mice showed a significantly higher number of sniffing bouts, nearly twice the number of bouts seen in the DE-exposed females (# *p* < 0.05; Two-way repeated-measures ANOVA, with Bonferroni correction). Habituation response was measured as a decreasing number of sniffing responses with repeated presentations of the same odor. DE-exposed mice of both sexes were unable to habituate to repeated presentation of same-sex urine, a social odor, while FA-exposed control mice of both sexes showed significant habituation toward same-sex urine. (*p < 0.05; **p < 0.01; ***p < 0.001; One-way repeated-measures ANOVA, with Dunn’s multiple comparison test). Graph plotted as mean ± SEM; FA M *n* = 9, DE M *n* = 10, FA F *n* = 8, DE F *n* = 7

In the olfactory habituation test (Fig. 7a, b), olfactory communication was assessed by quantifying sniffing responses elicited by repeated presentations of two non-social olfactory cues (almond and banana) and a social olfactory cue (sex-matched urine). During the first presentation of same-sex urine, DE-exposed female mice (Fig. 7b), but not males (Fig. 7a), showed a significant decrease in sniffing response compared to the FA-exposed animals. A habituation response in the olfactory test was reflected as a decreasing level of interest in subsequent repeated presentations of the same odor. Repeated presentation of non-social olfactory cues elicited a significant habituation response in all experimental groups, as expected, indicating unaffected olfactory function in the DE-exposed mice (Fig. 7a, b). DE-exposed mice of both sexes failed to habituate to the repeated presentation of same-sex urine, whereas FA-exposed males and females showed a significant habituation response (Fig. 7a, b).

### The effect of diesel exhaust on repetitive behavior

**Fig. 8:**
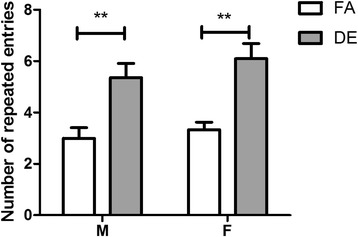
T-maze spontaneous alternation test. Number of repeated entries into the same arm was recorded in the T-maze spontaneous alternation test (mean ± SEM; *n* = 9–14). DE-exposed mice of both sex made significantly more repeated entries than FA-exposed mice of the same gender (**p < 0.01; Two-way ANOVA with Bonferroni correction)

In the T-maze spontaneous alternation test (Fig. 8), the number of repeated entries into the same goal arm by each mouse was scored as a measurement of repetitive behavior over 15 trials. DE-exposed mice of both sexes exhibited a significant increase in the number of repeated entries compared to FA-exposed mice of the same sex (Fig. 8). Mice that failed to choose a goal arm within the 2-min trial period were retested at a later time, and were removed from the test if they failed to choose a goal arm on the subsequent trial. A total of 6 mice (1 FA male, 2 FA females, and 3 DE females) were excluded using this criterion.

**Fig. 9:**
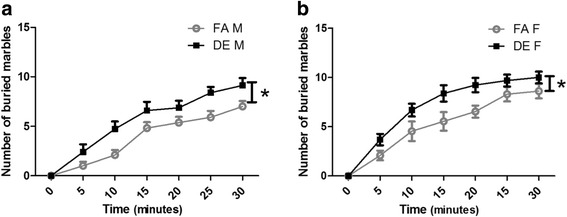
Marble Burying Test. For the marble burying test, the number of buried marbles was scored in 5 min intervals. Number of marbles buried by males (**a**) and females (**b**) were plotted separately (mean ± SEM; *n* = 11–15). DE-exposed mice of both sexes buried more marbles than FA-exposed control mice (Female *p = 0.0250; Male *p = 0.0263; Two-way repeated-measures ANOVA with Bonferroni correction)

The marble burying test (Fig. 9a, b) also demonstrated increased repetitive behavior associated with developmental DE exposure. For this test, the number of buried marbles was counted over a 5-min period as a proxy measurement for repetitive digging. In both sexes, DE-exposed mice buried more marbles than FA-exposed control mice of the same sex (Fig. 9a, b).

## Discussion

To our knowledge, this is the first detailed and comprehensive assessment of behavioral phenotypes relevant to all three characteristic domains of autism (difficulties in social interaction, communication deficiency, and increased repetitive behaviors) in mice exposed developmentally to an environmentally relevant level of DE (250–300 μg/m^3^). The level of DE exposure used in the current study falls into the “very unhealthy” and “hazardous” air quality index (AQI) categories [46]. These levels are not uncommon in cities such as Beijing, New Delhi, and Mexico City. Based on air quality data collected between April 2008 and March 2014 from a monitor installed at the U.S. Embassy in Beijing, air quality fell within the range of our exposure level about 20% of the total 2028 days surveyed [47]. Satellite monitoring data revealed that populations in some cities in South and Southeast Asia have been receiving prolonged exposure of > 100 μg/m^3^ of PM2.5 [17, 48].

Quality of maternal care has been known to have long-lasting effects on pup behavior; specifically, maternal neglect has been shown to lead to increased anxiety and hyperactivity behaviors [49]. The interpretation of behavioral testing results could be confounded by differences in locomotor activity, anxiety response, and other factors that may arise from these or other developmental defects. To assess the effects of DE exposure on maternal care behavior and subsequent consequence on pup anxiety, maternal care behavior and pup behavior in the open field test were assessed. No differences were observed in quality of maternal care or pup anxiety in the open field. Litter size, sex distribution, and pup weights did not differ between DE- and FA-exposed mice. Righting reflex also showed no differences between DE-exposed and FA-exposed animals. Altogether, these findings suggest that locomotor activity, anxiety, exploratory drive, and developmental deficits due to the maternal care environment played a minimal role, if any, in confounding the interpretation of behavioral testing results.

To assess the sociability domain, the three-chambered social preference test and the reciprocal interaction test were conducted. Exposure to DE did not affect sociability in the three chambered social preference test, as indicated by the preference of both DE-exposed mice and FA-exposed mice for the novel mouse over the empty chamber. However, in the social novelty phase, DE-exposed females showed no preference when presented with a choice between the novel and familiar mice, suggesting inability to differentiate social novelty. Inability in differentiating social novelty has also been reported in C3H/HeJ, AKR/J, A/J, and 129S1/SvImJ mouse strains, which have been reported to exhibit autism-like behavioral traits [50, 51]. Related to the fact that autism is a spectrum of disorders with a wide range of variation in severity and type of behavioral traits, we suspect that the inability to differentiate social novelty but not social preference, as shown in DE- exposed females, represents a more subtle but specific type of social deficit with recognizable functional implications. In the reciprocal interaction test, DE-exposed males exhibited significantly decreased social sniffing behaviors compared to FA-exposed males, and females showed a similar trend, though it was not statistically significant. Decreased male–male social sniffing has also been reported in the BTBR mouse strain and in Shank3+/− transgenic mice, which are genetic mouse models of ASD [52, 53]. Our results with these two tests suggest the presence of sex differences in the effect of DE on specific types of social behavior. While DE-exposed females exhibited a deficit in social novelty in the three-chambered social preference test, DE-exposed males exhibited a more robust phenotype in the reciprocal interaction test, where they showed a deficit in interactive sniffing episodes with same-sex mice. This difference in responding to social contexts provided by the two tests can likely be attributed to innate differences in behavioral sensitivity between male and female C57Bl/6J mice.

For the communication domain, neonatal isolation-induced USVs and olfactory habituation were used to assess vocal and olfactory communication, respectively. Mouse pups emit USVs with frequency ranges from 30 to 90 kHz [54]. Neonatal vocal repertoires similar to those observed in DE-exposed mice in the current study have also been detected in the reelin+/− and the BTBR T + tf/J mouse models of ASD during the early developmental period [42, 43, 55–58]. DE-exposed male pups showed a significant decrease in the total number of emitted USV calls, compared to FA males, and DE-exposed females showed a similar but not statistically-significant trend. A decreased number of USV calls has also been reported in the maternal immune activation (lipopolysaccharide treated) autism mouse model [58]. Sonographic patterns have been commonly analyzed in rodent models for detailed assessment of the types of calls emitted [41–44, 59, 60]. Nine call categories have been identified as the typical vocalization repertoire [41–44, 55, 56, 59–61] (See also Additional file 1: Figure S3); DE-exposed males and females emitted fewer calls of the frequency-step call category compared to FA-exposed mice. A similar shift in call-category preference has been reported in genetic autism models, such as the BTBR T + tf/J and reelin mutant mouse [44, 60], as well as mice exposed developmentally to chlorpyrifos [56]. In the current study, there was also a greatly increased number of unstructured USV calls in DE-exposed males and females. Unstructured calls are broken/deformed calls within the mouse pup’s frequency range, but which could not be recognized to fall in any of the nine call categories. Unstructured calls have also been reported in the BTBR T + tf/J mouse autism model [44]. While a large portion of the calls emitted by the DE-exposed mice in our study were unstructured calls, a few FA-exposed mice also produced unstructured calls, though they were very rare (1–2 unstructured calls in the 5 min recording session), and not all FA-exposed mice emitted them. Since naïve C57Bl/6 females have also been reported to produce small numbers of unstructured calls [44], unstructured calls in other published studies may have been present but overlooked, due to their rarity in control animals. In future studies, measuring unstructured USV calls could be a robust tool for assessing vocal communication deficits; however, additional research is needed to confirm that unstructured calls reflect a true communication deficit, as opposed to their representing an additional category of USV that is used for a specific type of communication.

In the olfactory habituation test, olfactory responses toward repeated presentations of social and non-social olfactory cues were measured; under typical situations, naïve mice would show decreasing interest with repeated presentations of the same scent, indicating habituation. The olfactory habituation test is also commonly used to assess olfactory communication in various autism mouse models [53, 62–64]. In our experiments, while mice from all experimental groups habituated to the repeated presentation of the two non-social odors (banana, almond); only FA-exposed mice were able to habituate toward social odors (sex- and age-matched pooled urine). These findings provide evidence that the inability of DE-exposed male and female mice to habituate to social odors is not due to loss of olfactory function, since DE-exposed animals of both sexes were able to habituate to non-social odors.

To assess the repetitive domain, the T-maze spontaneous alternation test and marble burying test were conducted. The T-maze has been used frequently to assess hippocampal dependent spatial memory under a variety of testing protocols [65–67]. In the T-maze spontaneous alternation protocol used in the current study, mice were allowed to explore freely within the T-maze and repetitive behavior was assessed by measuring repeated entries into the same goal arm in subsequent trials [65, 68–70]. Our data showed significant increases in the number of repeated entries by DE-exposed mice of both sexes, comparing to FA-exposed control mice; this result is consistent with findings reported in the maternal immune activation model and valproic acid-exposed model of autism [68, 70]. Repeated arm entries in the T-maze might be interpreted as a deficit in spatial working memory. However, our finding that repetitive behavior was also increased in the marble-burying test suggests that the T-maze results are best interpreted as an increase in repetitive behavior. The marble burying test has been used to assess repetitive behavior in rodent models of autism as well as in models of obsessive-compulsive disorder [71–76]. In the marble burying test, evenly spaced marbles serve as a proxy measurement for repetitive digging behavior; with increased repetitive digging more marbles will be buried. Our results show that DE-exposed males and females buried marbles more quickly and buried more marbles in the 30 min testing period, compared to FA-exposed control mice. As increased repetitive behavior in male and female DE-exposed mice were found in both of these independent tests, it is likely that developmental DE exposure affects repetitive behavior.

In recent years several large-scale epidemiological studies have shown associations between exposure to TRAP and increased risk of ASD [21, 23, 24, 77]. Associations between ASD related behavioral phenotypes and developmental ambient particulate exposures have also been recently assessed in different rodent models. A study conducted by Li et al. [78] in Sprague-Dawley rats exposed by intranasal instillation to ambient PM2.5 (2 or 20 mg/kg body weight) from PND 8 to 22 revealed decreased number of neonatal USV calls and inability to differentiate social novelty in exposed rats, in agreement with our findings. However, this study did not find deficits in social olfactory communication or increased repetitive behaviors, a difference from what we found. While highlighting the importance of critical windows of susceptibility during development, the differences between our findings and those of Li et al. [78] suggest that exposure to a complex mixture of air pollution (such as DE), rather than solely to ambient particulates, may represent a more relevant exposure scenario. Indeed, while several studies have shown that particles may have neurotoxic properties [79–83], other components of DE may contribute to its developmental neurotoxicity. In the current study, mice were exposed to diluted DE produced by a generator engine operating under load, resulting in DE with a physicochemical composition that included both particulate and gas components in a complex mixture. While the PM (and in particular the ultrafine PM) is a likely candidate for producing the effects on ASD-related behaviors, it is possible that nitrogen oxides or other gaseous components contributed to the effects, or that there was interactive toxicity among the various components. Another recent study by Church et al. [84] in B6C3F1 mice exposed to ambient PM2.5 (135.8 mg/m^3^) during the whole gestational period and up to PND10 found a behavioral phenotype similar to what we reported in the present study, e.g. a decreased interactive sniffing response, and increased repetitive grooming behavior. Exposure of mice to concentrated ambient ultrafine particles (UFP) during development caused lateral ventricle dilation, a predictor of poor neurodevelopmental outcome that has been associated with autism and schizophrenia [10, 35]. Prenatal DE exposure also had effects on motor coordination, impulsive behavior and monoaminergic systems in various brain regions in mouse models [36, 85].

In the present study, DE exposure was conducted throughout gestation and the pre-weaning period. This exposure period was chosen based on epidemiological studies in humans showing that traffic-related air pollution exposure during all three trimesters of pregnancy and during the first 9 months of infants’ life is associated with increased ASD risk [5, 22, 24, 27]. In mice, the neurodevelopmental period of this window of susceptibility equates to E0 to PND21 [33]. The current study does not address the relative importance of DE exposure during the gestational versus postnatal periods for producing effects on ASD-related behaviors. It would be of great interest to better define windows of DE susceptibility during development, and to ascertain mechanisms of specific CNS effects that occur during specific developmental events. Of some relevance to this are our unpublished findings from a preliminary pilot study that mice exposed only during the gestational period tended not to show effects on social behavior, repetitive behavior in the Morris Water Maze reversal task, or other neurobehavioral measures.

It is not clear whether the neurobehavioral consequences of DE exposure were due to direct effects on the CNS, e.g., by translocation of PM to the olfactory bulb, or to indirect effects arising from ingestion of DE particles or from peripheral inflammation, e.g., of the lungs. Indeed, developmental DE exposure of rodents has been shown to increase levels of pro-inflammatory cytokines in placenta, fetal brain and fetal lung, including studies done in the same facility and under the same conditions used in the current study [85–88]. In the current study, the route of exposure to DE clearly changed once the pups were born, as they began to breathe on their own and to inhale the DE directly, as well as possibly ingesting particles while nursing or grooming. Our group previously reported that acute DE exposure in adult mice causes neuroinflammation and oxidative stress in multiple brain regions [88]. DE particles, a primary component of TRAP, have also been reported to activate microglia both in vitro and in vivo [6, 89–91]. Further, developmental exposure to DE particles has been shown to increase inflammatory cytokines and alter microglia morphology in a toll-like receptor 4 (TLR4) dependent manner [31]. TLR4 has been shown to play a critical role in the maternal immune activation mouse model of autism, in which prenatal infection-induced activation of TLR4, and subsequent elevation of the key inflammatory cytokines IL6 and IL17α, are responsible for autistic-like behavior observed in pups [12, 91–93]. These findings suggest that DE exposure during perinatal development may activate pathways involved in maternal immune activation. The heterogeneous nature and escalating prevalence of ASD presents a complex and pressing public health challenge that warrants further mechanistic studies.

## Conclusions

The present study provides a comprehensive assessment of autism-related behavioral endpoints following developmental exposure to DE. Mice exposed to 250–300 μg/m^3^ DE from E0 to PND21 exhibited behavioral phenotypes consistent with all three characteristic domains of autism, i.e. increased repetitive behavior, altered vocal and olfactory communication, decreased social sniffing and inability to differentiate social novelty. Results from this study support epidemiological findings of an association between TRAP and ASD. Further mechanistic research in elucidating the mode of neurodevelopmental toxicity of DE is thus warranted.

## Additional files

Additional file 1 **Figure S1.** Three chambered social preference test set up. **Figure S2.** Open Field Test. **Figure S3.** Pup USV call categories. (DOCX 688 kb)

## Abbreviations

AQI: Air Quality Index
ASD: Autism spectrum disorders
DE: Diesel exhaust
E0: Embryonic day zero
FA: Filtered air
MIA: Maternal immune activation
PM: Particulate matter
PND: Postnatal day
TLR4: Toll-like receptor 4
UFP: Ultrafine particle
USV: Ultrasonic vocalization
