# Supplementary material for: Prenatal and early-life diesel exhaust exposure causes autism-like behavioral changes in mice
Source: Part Fibre Toxicol. 2018 Apr 20;15:18. doi: 10.1186/s12989-018-0254-4 (PMC5910592; doi:10.1186/s12989-018-0254-4)
Supplement: Supplementary file 1 — Figure S1. Three chambered social preference test set up. Figure S2. Open Field Test. Figure S3. Pup USV call categories. (DOCX 688 kb) [file 12989_2018_254_MOESM1_ESM.docx]

**Figure S1 Three chambered social preference test set up**

1a


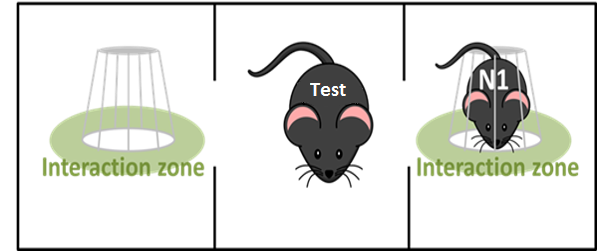


1b


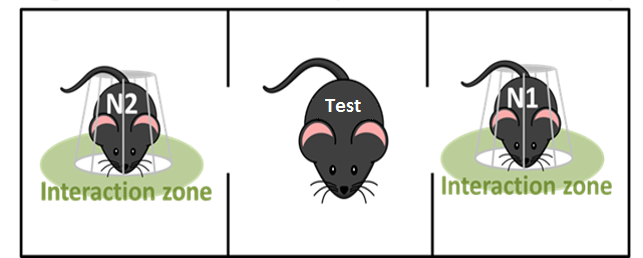


**A**. Sociability phase set-up: to test for sociability, one sex and age matched novel mouse (N1) was randomly placed into a metal holding cup while the test mouse was allowed to explore all three chambers freely. **B.** Social-novelty phase set-up: after the sociability phase an additional sex and age matched novel mouse (N2) was placed in the empty metal holding cup while the test mouse was allowed to explore all three chambers freely.

**Figure S2 Open Field Test**

**2a**


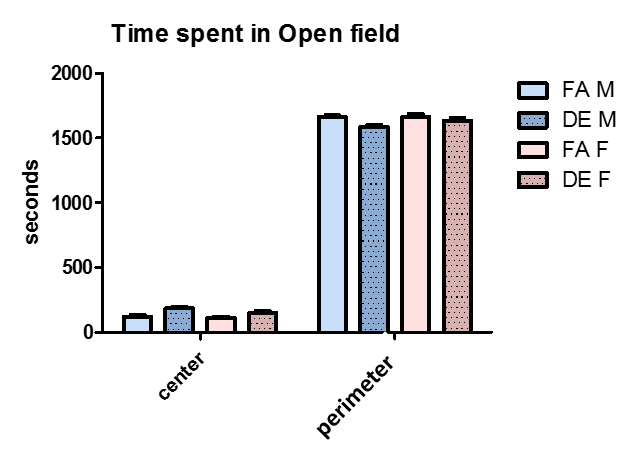


**2b**


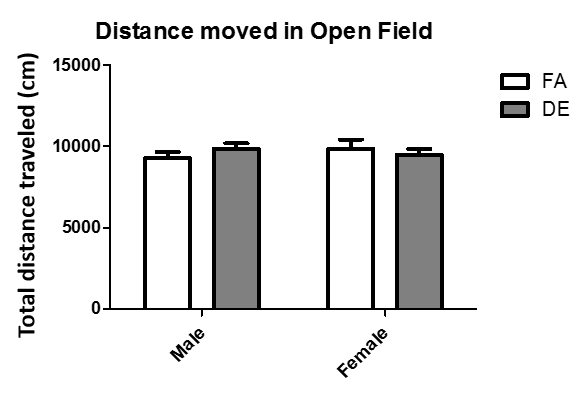


In the open field test, anxiety response was assessed by time spent in center vs. perimeter of the open field (A). Locomotor activity was measured by total distance traveled during the open field test (B). No significant differences were found in both assessments. FA M n=13, DE M n=14, FA F n=12, DE F n=13

**Figure S3 Pup USV call categories**


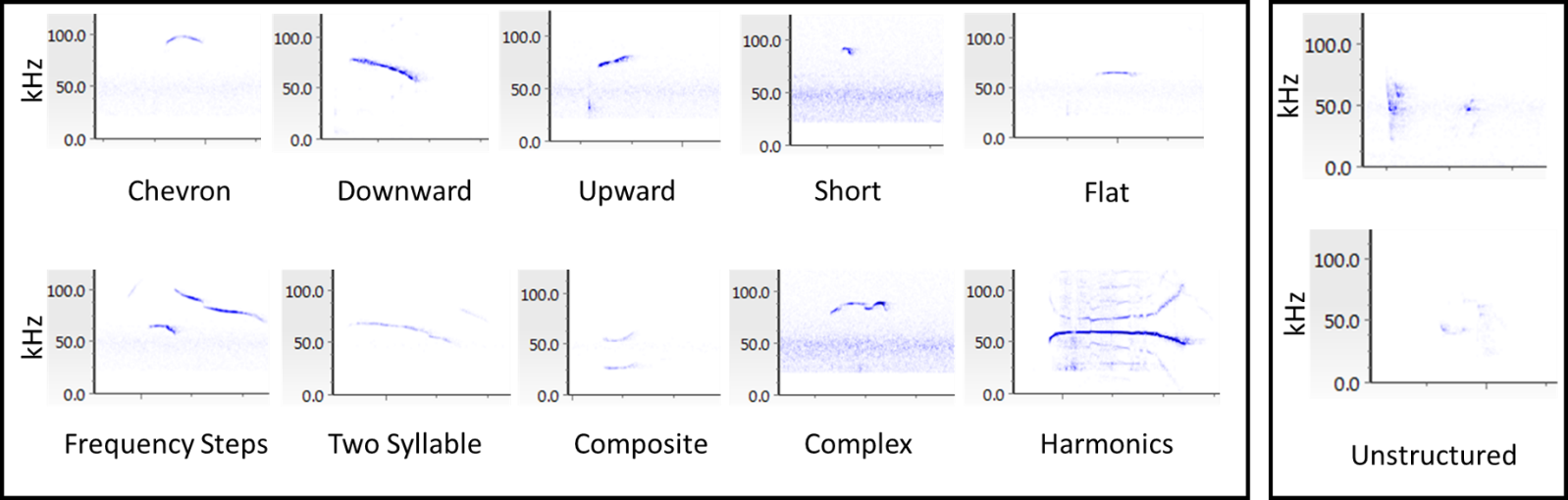


Representative call patterns of all nine call categories are depicted in supplemental figure 3. In the left panel two examples of unstructured calls emitted by DE exposed pups are provided. All USV calls depicted here were emitted by PND6 pups.
